# Options for Prospective Meta-Analysis and Introduction of Registration-Based Prospective Meta-Analysis

**DOI:** 10.3389/fpsyg.2016.02030

**Published:** 2017-01-04

**Authors:** Caroline A. Watt, James E. Kennedy

**Affiliations:** ^1^School of Philosophy, Psychology, and Language Sciences, University of EdinburghEdinburgh, UK; ^2^Independent ResearcherBroomfield, CO, USA

**Keywords:** meta-analysis, prospective meta-analysis, replication, study registration, researcher bias, confirmatory research

Many psychological researchers have recently recognized the need for preregistered, well-powered confirmatory studies (Nosek et al., 2012; Wagenmakers et al., 2012; Open Science Collaboration, 2015; van't Veer and Giner-Sorolla, 2016). These practices should eliminate most undetected methodological biases or “questionable research practices” that can distort study findings (Ioannidis, 2005; Simmons et al., 2011; John et al., 2012; Kaplan and Irvin, 2015; Franco et al., 2016) and they allow researchers to document verifiably that they used good methodology.

The present paper points out that the principles of preregistered, well-powered confirmatory research apply for meta-analyses as well as for individual studies. Typical retrospective meta-analyses resemble exploratory rather than confirmatory research. Decisions about studies to be included, statistical analyses, and moderating factors are made after the analysts know the outcomes of the studies. These retrospective decisions provide high potential for bias. Those wishing to challenge the findings of a retrospective meta-analysis easily find methodological decisions to debate. As Ferguson and Heene (2012) commented:

[W]e have seldom seen a meta-analysis resolve a controversial debate in a field.… [W]e observe that the notion that meta-analyses are arbiters of data-driven debates does not appear to hold true.… [M]eta-analyses may be used in such debates to essentially confound the process of replication and falsification. (p. 558).

The fundamental issue is that retrospective meta-analyses are a form of *post-hoc* analysis and *post-hoc* analyses are not effective at resolving scientific controversies. The extensive, prolonged, unresolved debates about meta-analyses in parapsychology clearly demonstrate this point (Honorton, 1985; Hyman, 1985, 2010; Milton, 1999; Storm, 2000; Bösch et al., 2006a,b; Radin et al., 2006; Storm et al., 2010a,b; Kennedy, 2013). The topics of debate have included the analysts' decisions about outcome measures, statistical methods, moderating variables, and inclusion/exclusion criteria for a meta-analysis.

To provide confirmatory evidence, the methodological decisions in a meta-analysis must be made prospectively, before the results of the included studies are known and ideally before the studies have been conducted. Three options for prospective meta-analysis are discussed below.

## Option 1. preregistered meta-analysis plan

The most obvious option is to preregister the meta-analysis plan and include in the meta-analysis only studies conducted after the plan was registered. The meta-analysis plan would specify the statistical analyses and the criteria for deciding which studies are included. Subsequent studies that comply with the inclusion/exclusion criteria would be included in the meta-analysis.

Unfortunately, this option is likely to retain significant retrospective decision-making. A study may be conducted with a novel variation of the procedures or measurement methods. Or, it may compare a standard condition with a modified condition. For many areas of research, it is unlikely that inclusion/exclusion criteria can be prospectively specified in sufficient detail to anticipate all the variations that will occur. The debates about handling variations in the studies testing the efficacy of psychological treatments for bipolar disorder and schizophrenia are an example of this dilemma (Jauhar et al., 2014, 2016). The natural (and very strong) tendency will be to decide whether a variation should be included based on the results. If the results conform to those obtained with more standard conditions or match the analyst's expectations, the variation will be considered appropriate to include, but may be excluded otherwise. This is the type of retrospective decision that introduces bias in a meta-analysis—and makes retrospective specification of inclusion/exclusion criteria much easier than prospective specification.

The use of preregistered meta-analysis plans is a substantial improvement over the typical retrospective meta-analysis and may be useful in some situations. However, it will not be optimal in many situations, particularly for controversial areas of research.

## Option 2. preplanned studies in a meta-analysis

A stronger option for prospective meta-analysis is to pre-specify the protocols for the included studies as part of the meta-analysis plan. The prospective meta-analysis becomes a large preplanned multi-center research project. The discussions and guidelines for prospective meta-analysis in medical research are based on this type of large project (Ghersi et al., 2011).

This type of prospective meta-analysis is similar to a large multi-center trial. Ghersi et al. (2011) note that prospective meta-analysis can allow individual researchers more autonomy over how they conduct their study locally. In multi-center trials, all investigators are typically required to adopt identical protocols. The investigators are essentially working as research assistants or co-experimenters under the guidance of the principal investigator(s). The greater autonomy of a prospective meta-analysis enables innovation and exploration of moderator variables that might not be permitted in multi-center trials. Also, the individual studies in a prospective meta-analysis are more likely to be published separately in addition to the meta-analysis, whereas the data collected at one site in a multi-center trial typically are not published separately from the results for the full study.

Ghersi et al. (2011) also note that more powerful multilevel modeling statistical methods can be used in this type of prospective meta-analysis because the raw data are available. Traditional meta-analysis methods were developed for situations when summary results are available but not the raw data.

Large multi-center research projects are the optimal strategy for obtaining scientific evidence, but unfortunately are often not possible in the behavioral sciences, which typically do not have the level of funding that is found in medical research. An alternative strategy for prospective meta-analysis is needed for wide use in behavioral science research—a strategy that is less dependent on big science.

## Option 3. registration-based prospective meta-analysis

We propose *registration-based prospective meta-analysis*, in which the decision to include or exclude a particular study is made prospectively based on the preregistration for the study. A study that will be included in the meta-analysis must be registered before data collection begins for the study. The studies will typically be independently initiated and funded, and not part of a large preplanned research effort associated with the meta-analysis. The decision to include or exclude a study in the meta-analysis will be made shortly after the study is registered and typically before data collection starts for the study. In effect, the registration for an individual study is used to prevent bias in a subsequent meta-analysis as well as to prevent bias in the individual study.

The specific steps for a registration-based prospective meta-analysis are: (a) the meta-analysis is planned, including power analysis, sample size, specific statistical methods, and criteria for including and excluding studies; (b) the meta-analysis plan is publicly preregistered; (c) a list of included studies is publicly maintained associated with the registration; (d) when a relevant study is registered, the registration is reviewed and a decision is made whether to include the study in the meta-analysis; (e) studies to be included are entered on the list of included studies; and (f) when the pre-specified sample size or other criteria for concluding the meta-analysis is reached, the meta-analysis is completed.

The list of included studies may have qualifications for the inclusion of data from a study. Any qualification will be prospectively specified at the time the study is added to the list. For example, for an experiment comparing a standard test condition with a modified test condition, the analysts may specify that the data from the modified test condition will be excluded from the primary confirmatory analysis. These pre-specifications assure that methodological decisions are made prospectively, while also allowing flexibility to adapt the meta-analysis to the unique characteristics of a study and not requiring that the meta-analysis plan anticipates all possible research variations.

A registration-based prospective meta-analysis can, and arguably should, be established for any line of research that has matured to the point of confirmatory studies. Ideally, a line of research will have at most one retrospective meta-analysis, and subsequent meta-analyses will be prospective. Like typical retrospective meta-analyses, a registration-based prospective meta-analysis does not control the research efforts. It simply specifies prospectively the methodological decisions for a subsequent meta-analysis.

Registration-based prospective meta-analysis is dependent on wide use of good study registration practices. It will not be viable if studies are not preregistered or are registered with some of the non-optimal registration processes that are currently available (Watt and Kennedy, 2015, also see the comments for that online article). We expect increasing development and use of optimal registration practices. A useful goal for study registries is to make registration-based prospective meta-analysis possible.

## Exemplar: a prospective meta-analysis for ganzfeld studies

As noted above, retrospective meta-analyses have failed to resolve the debates about hypothesized extra-sensory perception (ESP) abilities. As an exemplar for how prospective meta-analysis can be conducted, the first author has initiated a registration-based prospective meta-analysis for ESP studies employing the so-called ganzfeld method. The registration document is online at the KPU Study Registry (Watt, 2016). The details of the meta-analysis plan are available in the registration document and will not be repeated here. However, key points that are generally applicable for prospective meta-analyses will be noted.

Draft versions of the meta-analysis registration were widely circulated for comments by both proponents of ESP and skeptics. The goal of this peer review was to minimize avoidable controversy, not to achieve universal agreement. Significant improvements resulted from comments. We recommend that prospective meta-analysis have wide review prior to implementation.

The registration specifies the statistical methods that will test for overall ESP and that this is a confirmatory analysis. The guidance for the KPU Study Registry (2015) states that a well-designed confirmatory analysis should be capable of providing evidence that an experimental hypothesis is false as well as true and that all analysis decisions that could affect the results should be made before data collection starts. This requires that a confirmatory analysis has good statistical power and that the study uses established methods (Watt and Kennedy, 2015). A non-significant result for a study with high power is evidence that the hypothesis is false for the predicted effect size specified in the power analysis. Two analyses will evaluate the evidence for ESP and a correction for multiple analyses is specified.

The registration for the meta-analysis specifies the overall sample size that will be obtained based on a power of 0.95 for reasonable assumptions from previous studies. The meta-analysis will continue until that sample size is obtained. An option to complete the meta-analysis at a certain time point if the planned sample size was not yet obtained was initially proposed, but was rejected. Experimenters will typically know that a prospective meta-analysis is being conducted and will be able to track the outcomes of the studies included in the meta-analysis. If the initial studies are favorable, the experimenters may not conduct additional studies if they know that the meta-analysis will be completed on a certain date with whatever data are available. Other scenarios for bias can be imagined given the open tracking of progress for the meta-analysis. A fixed sample size provides the most unequivocal results.

The registration also specifies how protocol deviations will be handled, such as studies that are not completed. If protocol deviations occur, the analyses will be conducted with two steps. The first step will be an analysis that includes all of the available data for all the studies that were planned to be included. If this analysis gives significant results, a second analysis will be done that applies the principle of handling protocol deviations conservatively—with the assumptions that the experimental effect does not occur and that potential methodological biases from protocol deviations did occur. The registration document specifies how this will be done. If the first analysis gives significant results but the conservative analysis for protocol deviations does not give significant results, the meta-analysis will be considered to have produced inconclusive results due to protocol deviations.

## Additional methodological issues

Although prospective meta-analysis of preregistered studies eliminates the great majority of methodological issues that psychological researchers have been discussing recently, there are other significant methodological factors that it does not address. These factors have generally not yet been recognized by psychological researchers, but will eventually need to be addressed. These methodological factors include: software validation, measures to prevent or detect experimenter fraud, and appropriate statistical methods for confirmatory research. Kennedy (2016) provides practical recommendations for addressing these factors.

## Author contributions

Both authors have made substantial, direct and intellectual contribution to the work, and approved it for publication.

### Conflict of interest statement

The authors declare that the research was conducted in the absence of any commercial or financial relationships that could be construed as a potential conflict of interest.
